# Efficacy of sound therapy interventions for tinnitus management: A protocol for systematic review and network meta-analysis

**DOI:** 10.1097/MD.0000000000027509

**Published:** 2021-10-15

**Authors:** Hui Liu, Jin Zhang, Shuangyuan Yang, Xin Wang, Wen Zhang, Jiaying Li, Ting Yang

**Affiliations:** a Department of Otolaryngology, Shaanxi Provincial People's Hospital, China; b Xi’an Medical University, China.

**Keywords:** network meta-analysis, sound therapy, systematic review, tinnitus

## Abstract

**Background:**

Tinnitus is a common otological symptom and can be debilitating. Sound therapy has increased in popularity due to its potential for increased efficacy and fewer and milder side effects, but the available evidence is limited by the lack of randomized controlled trials comparing different sound therapies for tinnitus. Network meta-analysis (NMA) is a useful tool to compare multiple treatments when there is limited or no direct evidence available. The aim of this paper is to evaluate the efficacy and acceptability of different sound therapies for tinnitus.

**Methods and analysis:**

A literature search was conducted to identify articles in EMBASE, PubMed/MEDLINE, Web of Science, Cochrane Library, China National Knowledge Infrastructure, Chinese Biomedical Literature, and Wanfang and Weipu from inception to April 1, 2021. The Tinnitus Handicap Inventory, Tinnitus Questionnaire, and effective rate were used to assess perceived tinnitus suppression after treatment. We used Review Manager 5.4 for the standard meta-analysis; R 4.0.4 and Stata 15.1 were used for the NMA and the publication bias and sensitivity analyses.

**Results:**

The effect estimates of the direct comparisons (when available) were very similar to those of the NMA. Overall, sound stimulation alone performed better than medication alone, educational consultation alone, and no treatment. Combination therapy, such as sound stimulation plus educational consultation and sound stimulation plus drug therapy, yielded significantly better outcomes with regard to the alleviation of tinnitus than individual treatments.

**Conclusion:**

This is the first NMA to evaluate and compare the effectiveness of different sound therapies for the management of tinnitus. It may help inform the selection of sound therapy and the development of guidelines in clinical practice. Future studies of sound therapy with larger sample sizes involving multiple medical centers are needed to improve the current evidence.

## 1 Introduction

Tinnitus is a common otological symptom and is defined as a sound in the head or ears that has no external source.^[1–5]^ The sensation is usually defined as a ringing, buzzing, or whistling sound.^[1]^ Most people suffer from tinnitus at least once in their life and approximately 1% to 6% of the population is severely affected by tinnitus, experiencing sleep disorders, headaches, weakness, depression, and confusion or difficulty concentrating.^[6]^ However, the pathophysiology of tinnitus remains unclear, and because otological conditions, especially high-frequency hearing loss, are major risk factors for tinnitus, the sensations are often deemed to be a maladaptive homeostatic compensation mechanism that is triggered by auditory deprivation.^[5]^ Treatments to reduce tinnitus and tinnitus-related distress include auditory therapeutic measures, repetitive transcranial magnetic stimulation, direct current stimulation, specific forms of acoustic stimulation (noise/masking, retraining therapy, music, and acoustic coordinated reset), laser treatment, acupuncture, surgery, and so on. However, no specific treatments or interventions have yet been proven to offer a completely satisfactory solution.^[7–9]^

Acoustic therapy was first described in a medical textbook by Jean-Marie Itard in 1821, and this treatment continues to evolve, with a range of therapies being developed^[10]^; therapies include tinnitus retraining therapy (TRT) (sound stimulation combined with educational consultation), hearing aids (HA), masking and customized sound therapy, which is a tinnitus management strategy based on the individual's condition, that includes personalized notched music training, tinnitus pitch-matched therapy, neuromonics tinnitus therapy, and so on.^[7,11–13]^ Sound therapy is one of the most common methods of managing tinnitus and its effectiveness with regard to changing a patient's perception of tinnitus has been explored for centuries. Because of its noninvasiveness and simplicity, sound therapy is readily accepted by patients and widely used in clinical practice.^[7]^ However, reports of its efficacy have been mixed.

In light of these issues, the present study aimed to systematically review the current literature on sound therapy and explore the true effect of sound therapy. We sought to comprehensively assess the effect size by conducting a network meta-analysis (NMA) of published studies in Chinese and English, as sound therapy may represent a promising strategy with which to suppress tinnitus.

## 2 Materials and methods

### 2.1 Systematic search strategy and study selection

Two investigators (YT and LH) independently searched for articles without language restrictions from the date of the inception of each database through April 1, 2021. The databases that were searched were EMBASE, PubMed/MEDLINE, Web of Science, Cochrane Library, China National Knowledge Infrastructure, Chinese Biomedical Literature, Wanfang, and Weipu databases. Medical subject heading and free search terms were both used in the literature search. An example of the search strategy for PubMed/MEDLINE is (“tinnitus” AND (“masking therapy” OR “tinnitus retraining therapy” OR “hearing aids” OR “music therapy” OR “sound therapy” OR “acoustic therapy”) AND (“randomized controlled trial” OR “RCT” OR “randomized”)). The Preferred Reporting Items for Systematic Reviews and Meta-Analysis (PRISMA) statement and checklist were followed as much as possible during this review,^[14]^ and the protocol was reviewed and registered in PROSPERO (ID: 4202159034). We initially identified 863 articles after reading the titles and abstracts.

Two investigators (YT and ZJ) independently skimmed the identified abstracts and selected the articles for the full-text review. The same investigators independently reviewed the full texts of the selected articles. A senior investigator (LH) adjudicated in cases of disagreement.

The studies were identified using the PICOS (participants, interventions, comparators, outcomes, and study designs) framework to set the parameters of interest. These can be summarized as follows: (P) Patients with acute or chronic tinnitus. (I) Sound stimulation alone, sound stimulation combined with drug therapy, sound stimulation combined with educational consultation, or sound stimulation combined with drug therapy and educational consultation. (C) Drug therapy, no treatment, educational consultation only, sound stimulation alone or sound stimulation combined with drug therapy (O) The clinical outcomes of the studies included the Tinnitus Handicap Inventory (THI), Tinnitus Questionnaire (TQ), and effective rate. (S) RCTs.

Studies with the following characteristics were excluded: (a) a study design other than an RCT; (b) not in English or Chinese; (c) only 1 arm; (d) missing key information, such as a suitable comparator and the main quantitative outcomes; (e) animal experimental investigations, case reports, meeting abstracts and comments, and review articles; (f) unavailable data; (g) incomplete or seriously flawed studies; and (h) not the largest study among those using duplicate patients within the same institution.

### 2.2 Quality assessment

The Cochrane Collaboration tool was used to assess the risk of bias in the selected studies. The following aspects were assessed independently by 2 reviewers (YT and YSY): random sequence generation, allocation concealment, blinding of participants and personnel, blinding of outcome assessment, incomplete outcome data, selective reporting, and other bias. Disagreements between the 2 reviewers were resolved through either discussion or adjudication by a third reviewer (LH). We judged each study as having a low, unclear, or high risk of bias in each domain. In the review of randomization, studies that described their exact randomization method were scored as having a low risk of bias; however, when the study did not report the exact randomization method but indicated that they had randomized, controlled designs, we scored them as “unclear.” Similar criteria were applied for the scoring of allocation concealment, blinding, incomplete outcome data, and selective reporting.

### 2.3 Statistical analysis

Review Manager 5.4 (Cochrane), Stata 15.1, and R 4.0.4 were used for statistical analysis.

#### 2.3.1 Pairwise meta-analysis

The effect estimate for the THI and TQ was the mean difference (MD) and the effective rate estimated by the rating scale scores was the odds ratio (OR). The MD and 95% confidence interval (CI) were calculated directly from data reported in figures or the main text. The Cochrane *Q* statistic and the *I*^2^ statistic were used to assess statistical heterogeneity. Heterogeneity was categorized as follows using the Nordic Cochrane Centre (2011) reference: *I*^2^ = 0% to 40%, no important heterogeneity; *I*^2^ = 30% to 60%, moderate heterogeneity; *I*^2^ = 50% to 90%, substantial heterogeneity; and *I*^2^ = 75% to 100%, considerable heterogeneity. If the *I*^2^ statistic was greater than 50% and the Cochrane *Q* – statistic had a *P* value <.1, a random-effects (RE) model was used. Otherwise, we used a fixed-effects model, namely, the Mantel–Haenszel method, to calculate the pooled effect. *P* < .05 was considered significant. Forest plots were generated to illustrate the study-specific effect sizes along with the corresponding 95% CI.

#### 2.3.2 Network meta-analysis

We performed Bayesian NMA to compare multiple sound therapies by combining direct and indirect evidence of the relative treatment effects. The NMA was conducted with R4.0.4. Considering the expected between-study heterogeneity, we used a RE model for each comparison. The consistency of the evidence was assessed by fitting unrelated means models and comparing their deviance information criteria (DIC) with that from the corresponding consistency model (with differences of 5 points or more indicating an important difference in fit). Meanwhile, the local inconsistency of our results was confirmed by the node-splitting method and its Bayesian *P* value. *P* values >.05 indicated good consistency among the reports.

Similarly, the effect estimates were calculated using ORs with 95% CIs for dichotomous data; continuous data are expressed as the SDs for each study, and the MDs with 95% CIs were calculated. We also compared the direct and indirect estimates for each comparison. Network plots were drawn to describe and present the geometry of the treatment network of comparisons across trials to ensure that NMA was feasible. Additionally, the relevant rank plots based on the probability for different endpoints are shown.

### 2.4 Sensitivity analyses

We also performed sensitivity analyses to evaluate the impact of both studies with high levels of risk of bias and those with various follow-up durations on our results. Sensitivity analyses were performed by excluding 1 paper at a time and observing the robustness of the results.

### 2.5 Publication bias

We used comparison-adjusted funnel plots to explore the potential small-study effects in the network and used contour-enhanced funnel plots to examine whether the funnel plot asymmetry was caused by publication bias.

### 2.6 Ethics and dissemination

Since existing studies are involved, there is no need for ethics approval.

## 3 Results

### 3.1 Search results and study characteristics

**Figure 1:**
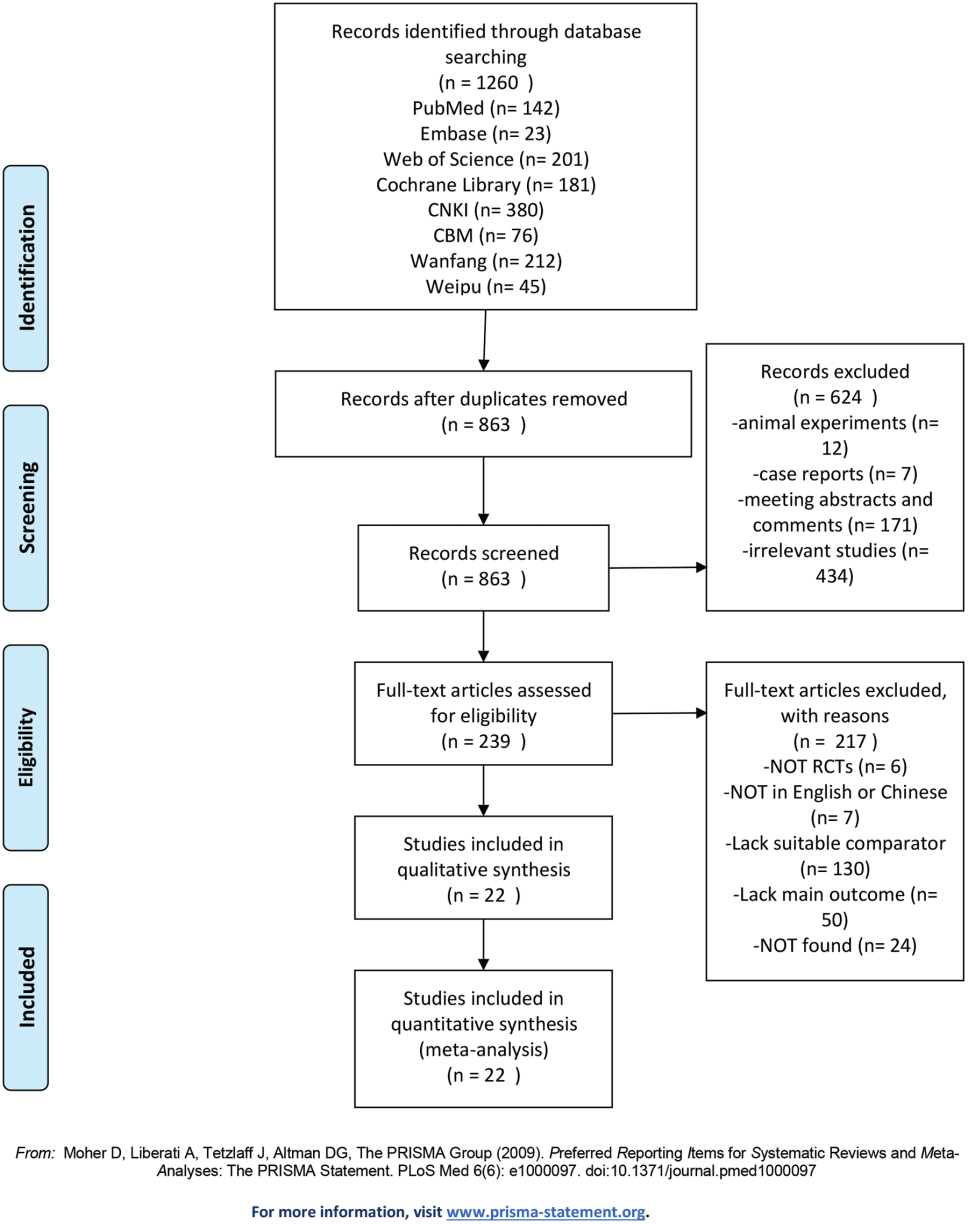
Flow diagram of the selection of the included studies.

A total of 1269 citations were identified from the EMBASE, PubMed/MEDLINE, Web of Science, Cochrane Library, China National Knowledge Infrastructure, Chinese Biomedical Literature, Wanfang, and Weipu databases. The number of citations decreased to 863 after duplicates were removed. By screening titles and abstracts, we excluded 624 studies for different reasons. Of the 239 potentially eligible studies, 217 articles were excluded, for the following reasons: they were not RCTs (n = 50) and full texts were not found (n = 24). Finally, 22 studies were included. Twenty studies with 1522 participants were included in this review. Seven studies were published in English and the remaining 15 studies^[17,19,20–23,25,26,28–31,34–36]^ were published in Chinese. The greatest number of studies originated in China,^[17,19,20–23,25,26,28–31,34–36]^ Germany,^[16]^ Sweden,^[18]^ the United States,^[24,27,33]^ and Brazil^[32]^ and were published from 2005 to 2020. The search strategy is shown in Figure 1 and descriptions and patient characteristics are summarized in Table 1. The treatment details in the selected studies are shown in Table 2.

**Table 1:** Charateristics of the included studies and participants.

			Sample size		Sex (M + F)		Tinnitus duration		Age	
Author (publication year)	Country	Types of tinnitus	E	C	E	C	E	C	E	C
Hiller and Haerkötter (2005)^[15]^	Germany	Chronic tinnitus	31	33	33 + 15	18 + 15	≥6 mo		52.5 ± 15.3	45.2 ± 14.1
			31	29	21 + 10	12 + 17			51.0 ± 13.2	51.4 ± 10.9
Caffier et al (2006)^[16]^	Germany	Chronic tinnitus	20	20	22 + 18		≥6 mo		51 yrs	
Wang et al (2010)^[17]^	China	Sensorineural tinnitus	35	30	16 + 19	13 + 17	2 d–5 yrs		19–70	21–67
Westin et al (2011)^[18]^	Sweden	Chronic tinnitus	20	20	12 + 8	6 + 14	9.19 ± 6.61	6.77 ± 5.95	48.95 ± 14.5	53.5 ± 12.84
				22		14 + 8		7.11 ± 7.73		49.59 ± 11.86
Deng et al (2012)^[19]^	China	Subjective tinnitus	292	395	378 + 309		≥3 mo		41.3	
Yang (2014)^[20]^	China	Sugjective tinnitus	68	73	42 + 26	48 + 25	2.7 ± 0.8 yrs	3.1 ± 0.3 yrs	55.4 ± 3.2	57.6 ± 2.9
Luo et al (2014)^[21]^	China	NA	40	40	18 + 22	18 + 22	2–5 yrs		19–68	21–67
Liu et al (2015)^[22]^	China	Sudden deafness with tinnitus	48	48	NA		<15 d		18–75 yrs	
Zhang (2015)^[23]^	China	NA	43	42	27 + 16	23 + 19	NA		50	50
Henry et al (2016)^[24]^	America	Chronic tinnitus	42	39	40 + 2	39 + 0	≥6 mo		62.4 ± 9.8	62.7 ± 10.6
			34	33	33 + 1	32 + 2			60.1 ± 10.1	61.2 ± 8.8
Wang et al (2017)^[25]^	China	Sudden deafness with tinnitus	68	56	39 + 29	23 + 33	≤14 d		47.44 ± 12.52	48.91 ± 10.15
Han (2017)^[26]^	China	Sensorineural tinnitus	30	30	17 + 13	18 + 12	40 d–50 yrs	30 d–16 yrs	43.11 ± 12.15	62.23 ± 3.28
Bauer et al (2017)^[27]^	America	Subjective, stable, bothersome chronic tinnitus	19	19	13 + 6	13 + 6	NA		NA	
Wang et al (2018)^[28]^	China	NA	30	30	17 + 13	16 + 14	3.5 ± 1.5	3.7 ± 1.3	52.1 ± 4.3	53.4 ± 3.9
Liu et al (2018)^[29]^	China	Sudden deafness with tinnitus	30	30	16 + 14	17 + 13	NA		40.98 ± 14.96	40.33 ± 15.42
He et al (2018)^[30]^	China	Subjective tinnitus	40	40	20 + 20	22 + 18	≥6 mo		42.5 ± 9.17	43.3 ± 12.27
Zhang (2019)^[31]^	China	NA	30	30	18–60		>5 yrs		48.23 ± 8.41	51.27 ± 9.02
Radunz et al (2019)^[32]^	Brazil	NA	11	11	NA		58.9 ± 17.7 mo		56.3 ± 16.8	
			11							
Scherer and Formby (2019)^[33]^	America	Subjective distress tinnitus	51	49	34 + 17	36 + 13	10.9 ± 8.9y	13.0 ± 11.4 yrs	51.1 ± 12.6	49.9 ± 10.0
			51		37 + 14		11.7 ± 11.1		50.9 ± 11.2	
Xiao (2020)^[34]^	China	Sensorineural tinnitus	33	33	19 + 14	20 + 13	30 d–16 yrs		41.27 ± 11.52	41.26 ± 11.31
Zhang et al (2020)^[35]^	China	Subjective tinnitus	59	59	21 + 38	20 + 39	2.27 ± 0.46	2.36 ± 0.47	40.27 ± 13.29	41.35 ± 14.27
Luo et al (2020)^[36]^	China	NA	30	30	22 + 38	5.9 ± 2.1	4.2 ± 3.8	48.2 ± 14.6	50.1 ± 12.7	

**Table 2:** Details of the treatment conditions.

	Intervention		Intervention time and frequence			
Author (publication year)	E	C	E	C	Outcome measures	Follow-up period
Hiller and Haerkötter (2005)^[15]^	TE with NG	TE without NG	Four 90-min weekly sessions with behind-the-ear (bte) broadband white NGs, 1 for each ear	Four 90-min weekly sessions without NG	TQ; T-Cog; VAS tinnitus loudness; VAS tinnitus unpleasantness; VAS control of tinnitus; WI; DAQ	18 mo
	CBT with NG	CBT without NG	Ten 120-min sessions with behind-the-ear (bte) broadband white NGs, 1 for each ear	Ten 120-min sessions without NG		
Caffier et al (2006)^[16]^	TCT with TCIs	TCT without TCI devices	Counseling, fitting patients with TCIs (TCI provision), auditory and relaxation training and psychosomatic care	Counseling, auditory and relaxation training and psychosomatic care without TCI	TQ; VAS loudness; VAS annoyance; VAS awareness	24 mo
Wang et al (2010)^[17]^	Sound information therapy	Drug therapy	Sound information therapy 10 consecutive daily sessions	Microcirculation and neurotrophic drug treatment	Effective rate	0
Westin et al (2011)^[18]^	TRT	ACT	Sound therapy and retraining counseling. Patients were given a 30 min follow-up session over telephone following the same principles. The treatment went on for 18 months in total	1 time weekly,a maximum of 10 sessions was offered and each session was set to be 60 min	THI; ISI; QOLI; HADS; CGI-I; TAQ	18 mo
		WLC		Waiting for treatment with no therapy		
Deng et al (2012)^[19]^	TRT	Drug therapy	Narrowband noise 2–3 times/d, 10 min 1 time for 1 month combined with counseling therapy	Expand blood vessels neurotrophic anxiolytic drug treatment	Effective rate	NA
Yang (2014)^[20]^	Sound therapy with drug treatment	Drug therapy	Sound therapy 1 h/d for 4–6 mo combined with taking Flunarizine orally	Flunarizine 10 mg every night, a 12 days of treatment course, 3 courses as total.	Effective rate	NA
Luo et al (2014)^[21]^	Sound information therapy with drug therapy	Drug therapy	Sound information therapy 1 time/d for 10 days as a course with drug therapy	Take Flunarizine, *Ginkgo biloba*, Betahistine, VitB orally	Effective rate	NA
Liu et al (2015)^[22]^	Composite acoustic therapy combined with drug therapy	Drug therapy	Acoustic therapy 2 times a day for 30 min with drug therapy	Methylprednisolone, Ginkgo-damole, and Caisch intravenous injection 1 time a day combined with taking Mecobalamin orally 3 times a day for 14 d	VAS, THI, SAS, Hearing recovery rate	3 mo
Zhang et al (2015)^[23]^	Sound therapy	Drug therapy	Sound therapy 30–45 min, twice a day, 4 weeks as a course for 2 courses	Flunarizine hydrochloride 10 mg with Clonazepam 5 mg once a day for 4 weeks	Effective rate	NA
Henry et al (2016)^[24]^	TM	TED	NA	NA	THI	18 mo
	TRT	WLC	NA	No treatment		
Wang et al (2017)^[25]^	TRT with drug therapy	Drug therapy	Masking, relaxation training, distracting, counseling, and drug therapy	NA	Effective rate	6 mo
Han (2017)^[26]^	Sound information therapy with drug therapy	Drug therapy	Sound information therapy once a day for 20–30 min with drug therapy	Compound Danshen, energy mixture and VitB6	Effective rate	NA
Bauer et al (2017)^[27]^	TRT	SC	Broadband noise and counseling	General aural rehabilitation counseling distributed over 3 1-h sessions, using a standardized SC Powerpoint presentation	THI, TFI, TIQ, TEQ	18 mo
Wang et al (2018)^[28]^	Sound therapy	Sound therapy with drug therapy	Sound stimulation 30 min twice a day for 3 mo	Sound stimulation 30 min twice a day for 3 mo with oral Chinese traditional medicine twice a day for 1 week	Effective rate	3 mo
Liu et al (2018)^[29]^	TRT with drug therapy	Drug therapy	Composite acoustic therapy, counseling combined with Methylprednisolone, Ginkgo-damole Alprostadil, and Caisch intravenous injection	Intratympanic Lidocaine injection every other day for 10 days combined with Methylprednisolone, Ginkgo-damole Alprostadil, and Caisch intravenous injection	Effective rate, GQOL-74	NA
He et al (2018)^[30]^	Sound therapy	Blank space group	Sound therapy once a day for 15 min, 10 times as a course for 3 courses	No treatment	THI, effective rate	1 mo
Zhang (2019)^[31]^	Sound therapy with educational consultation	Sound therapy	Sound therapy 30 min once a day for 1 m with online consultation	Sound therapy 30 min once a day for 1 mo	THI, effective rate	0
Radunz et al (2019)^[32]^	HA	Drug therapy	equipped with Beltone individual HA digital	Drug therapy with *G. biloba* extract EGb 761, dose of 240 mg/day/patient	THI, VAS	3 mo
	HA combined with drug therapy		Both drug therapy with *G. biloba*, and to HA			
Scherer and Formby (2019)^[33]^	TRT	SoC	TC and ST implemented with ear level SGs	Standard of care, a patient-centered tinnitus approach, focused on the individual participant's symptoms and aimed to reduce negative cognitive, affective, physical, and behavioral reactions to tinnitus	TQ, TFI, THI, VAS, sleep disturbance subscale, auditory difficulties subscale, relaxation interference subscale, reduced quality of life subscale	18 mo
	Partial TRT		TC and placebo SGs			
Xiao (2020)^[34]^	Sound information therapy with drug therapy	Drug therapy	Sound information therapy for 20 min once a day for 10 days	Microcirculation and neurotrophic drug treatment	Effective rate	NA
Zhang et al (2020)^[35]^	Sound therapy with drug therapy	Drug therapy	Sound therapy twice a day for 30 min for 3 mo	*G. biloba* 80 mg twice a day	Effective rate	NA
Luo et al (2020)^[36]^	Sound therapy with educational consultation	Sound therapy	Sound therapy 2 h for 3 mo with online consultation	Sound therapy 2h for 3 months	Effective rate	NA

### 3.2 Risk-of-bias assessment

**Figure 2:**
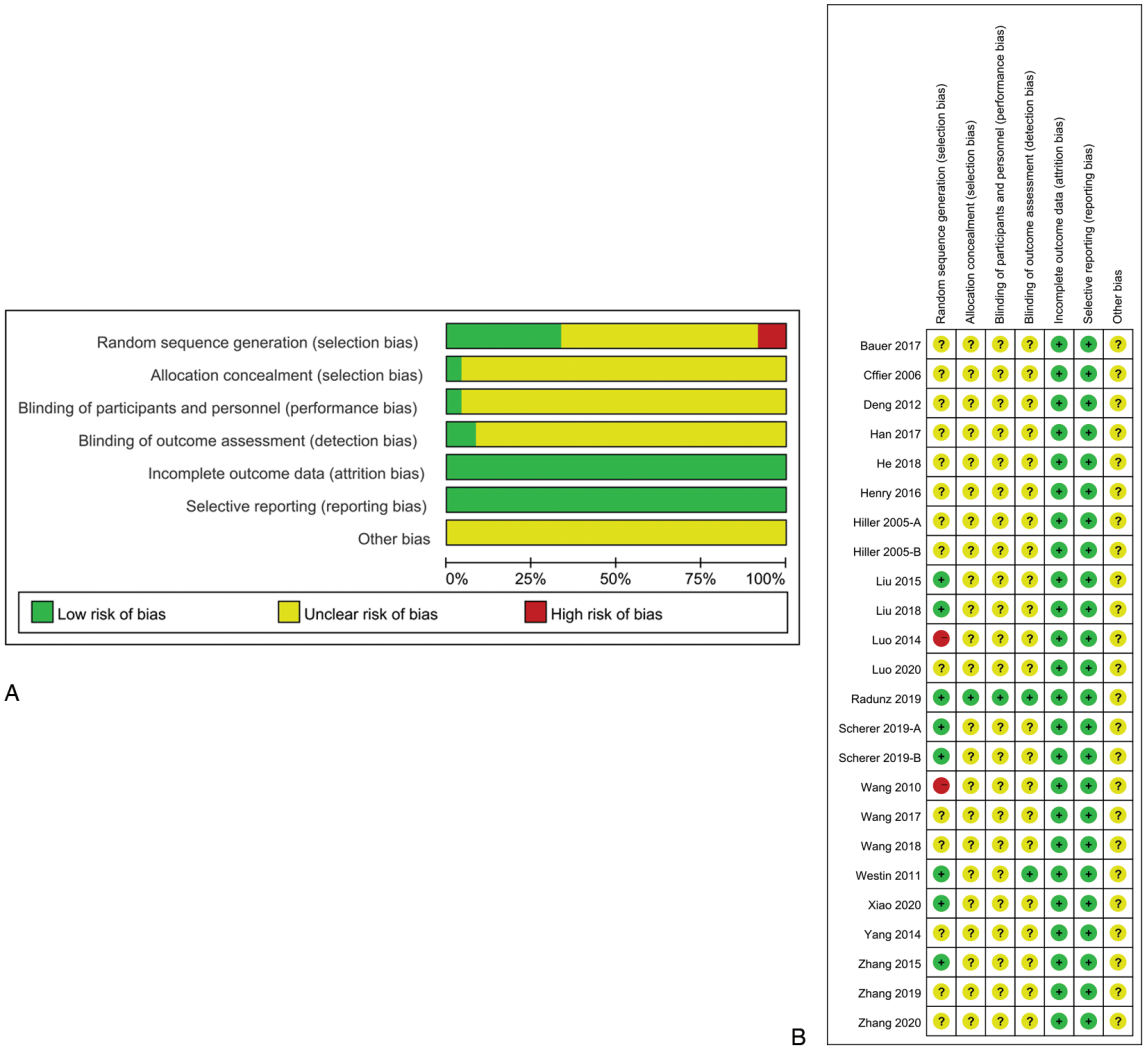
Risk-of-bias summary and graph. (A) Risk of bias graph and (B) Risk of bias summary.

All included studies mentioned randomization. Only 6 studies^[18,22,23,29,32–34]^ described the detailed methods of randomization. Only 1^[32]^ study clearly described the concealment of allocation. One^[32]^ study had an explicit double-blind design and 1^[18]^ study had a single-blind design. In the domain of incomplete outcome data and selective reporting, all the studies were judged as having a “low” risk of bias. Other biases sometimes included unknown risk, so we scored all the other biases as “unclear” (Fig. 2A,B).

### 3.3 Pairwise meta-analysis

#### 3.3.1 Tinnitus Handicap Inventory

##### 3.3.1.1 Sound stimulation vs no treatment

**Figure 3:**
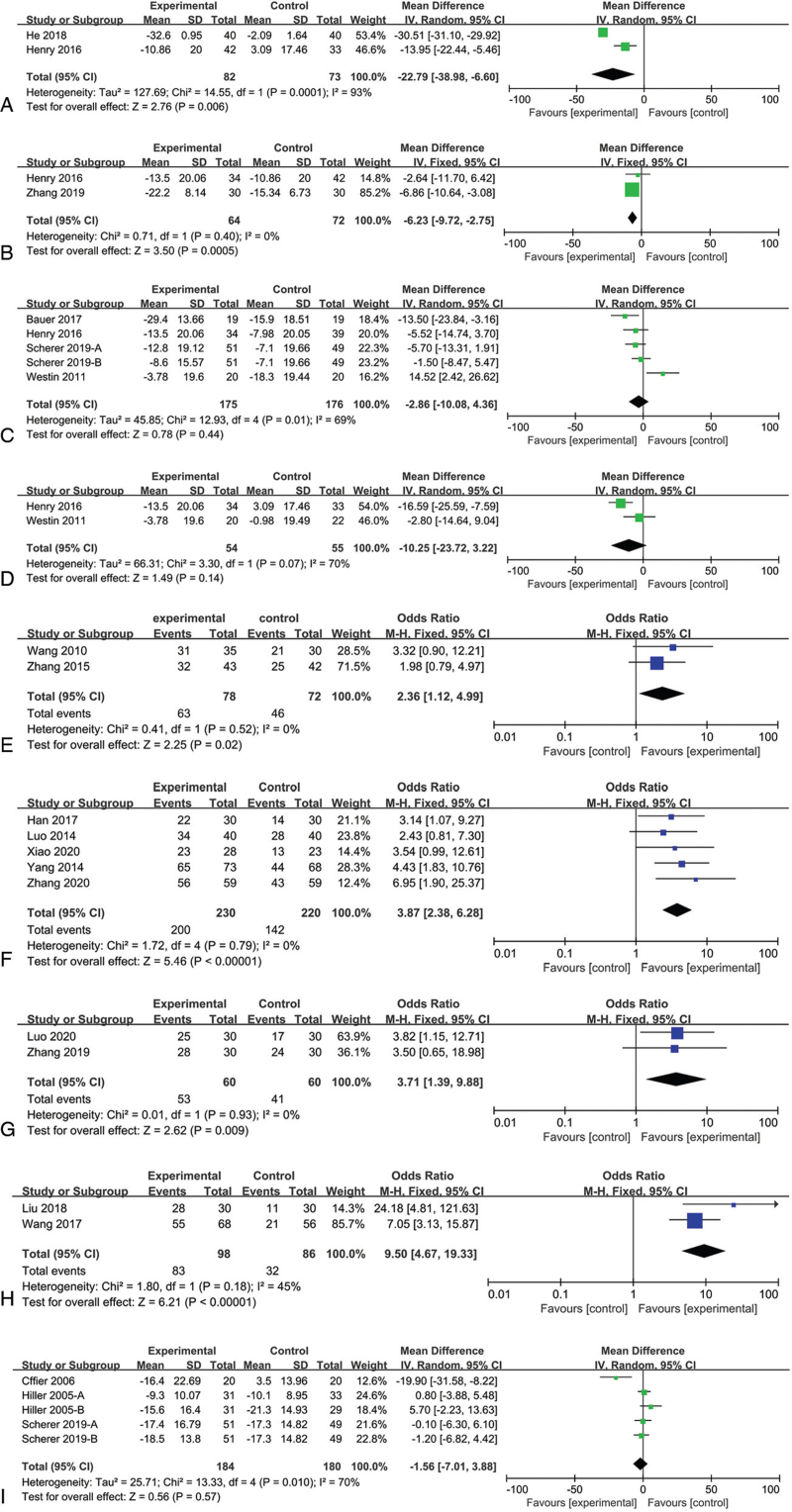
Meta-analysis forest plot of the effective rate, changes in the THI scores, and TQ score in tinnitus patients. (A) Sound stimulation versus no treatment for the THI change scale score; (B) Sound stimulation with educational consultation versus sound stimulation for the THI change scale score; (C) Sound stimulation with educational consultation versus educational consultation for the THI change scale score; (D) Sound stimulation with educational consultation versus no treatment for the THI change scale score; (E) Sound stimulation versus drug therapy for effective rate; (F) Sound stimulation with drug therapy versus drug therapy alone for effective rate; (G) Sound stimulation with educational consultation versus sound stimulation alone for effective rate; (H) Sound stimulation with educational consultation and drug therapy versus drug therapy alone for effective rate; and (I) Sound stimulation group versus the educational consultation for TQ change scale score. THI = Tinnitus Handicap Inventory, TQ = Tinnitus Questionnaire.

Two studies that compared sound stimulation with no treatment found a significant difference (MD, −22.79; 95% CI, −38.98, −6.60; *P* = .006) (Fig. 3A). We used an RE model as there was substantial heterogeneity among studies (*I*^2^ = 93%, *P* = .0001).

##### 3.3.1.2 Sound stimulation with educational consultation vs sound stimulation

Sound stimulation with educational consultation and sound stimulation alone significantly affected the THI scores (MD, −6.23; 95% CI, −9.72, −2.75; *P* = .0005), with no significant heterogeneity (*I*^2^ = 0%, *P* = .0005) (Fig. 3B).

##### 3.3.1.3 Sound stimulation with educational consultation vs educational consultation

Two studies provided data on the changes in the THI scores after sound stimulation with educational consultation and educational consultation alone (Fig. 3C). Pooled analysis of the data showed no significant improvement in THI scores (MD = −2.86; 95% CI, −10.08, 4.36; *P* = .44), and there was moderate heterogeneity between the studies (*I*^2^ = 69%, *P* = .01).

##### 3.3.1.4 Sound stimulation with educational consultation vs no treatment

As shown in Figure 3D, sound stimulation with educational consultation and no treatment did not yield significant differences in the THI score. The MD was −10.25 (95% CI, −23.72, 3.22; *I*^2^ = 70%).

#### 3.3.2 Effective rate

##### 3.3.2.1 Sound stimulation vs drug therapy

Two studies compared the efficacy of sound stimulation and drug therapy using the effective rate, and a fixed-effects model was used due to the lack of important heterogeneity (*I*^2^ = 0%, *P* = .52). The pooled analysis showed that sound stimulation yielded a significantly higher effective rate than drug therapy (OR, 2.36; 95% CI, 1.12, 4.99; *P* = .02) (Fig. 3E).

##### 3.3.2.2 Sound stimulation with drug therapy vs drug therapy alone

Five studies compared the efficacy of sound stimulation combined with drug therapy with that of drug therapy alone, and the pooled OR was 3.87 (95% CI, 2.38, 6.28; *P* < .00001), indicating a statistically significant difference between the 2 groups, a fixed-effects model was used due to the lack of important heterogeneity (*I*^2^ = 0%, *P* = .79) (Fig. 3F).

##### 3.3.2.3 Sound stimulation with educational consultation vs sound stimulation alone

Two studies with 120 participants compared the efficacy of sound stimulation with educational consultation with that of sound stimulation alone using the effective rate, and a fixed-effects model was used due to the lack of important heterogeneity (*I*^2^ = 0%, *P* = .93). The pooled analysis showed that sound stimulation combined with educational consultation yielded a significantly higher effect on effective rate than drug therapy (OR, 3.71; 95% CI, 1.39, 9.99; *P* = .009) (Fig. 3G).

##### 3.3.2.4 Sound stimulation with educational consultation and drug therapy vs drug therapy alone

Two studies with 286 participants compared sound stimulation with educational consultation and drug therapy with drug therapy alone using the effective rate. The results showed a significant overall effect, and the pooled analysis demonstrated that sound stimulation with educational consultation and drug therapy yielded a significantly higher effective rate than drug therapy (OR, 9.50; 95% CI, 4.57, 19.33; *P* < .00001; *I*^2^ = 45%) (Fig. 3H).

#### 3.3.3 Tinnitus questionnaire

To compare the change in distress caused by tinnitus according to the TQ score between the sound stimulation group and the educational consultation group, a random-effects model was used due to the presence of moderate heterogeneity (*P* = .01; *I*^2^ = 70%). The analysis showed no difference in the effects of the 2 regimens (MD, −1.56; 95% CI, −7.01, 3.88; *P* = .57) (Fig. 3I).

### 3.4 Network meta-analysis

#### 3.4.1 Network diagram

We compared all of the included studies with results assessed based on the THI and effective rate. Network diagrams were constructed incorporating the studies into quality-based displays on a network map that visually showed the distribution of the studies (Fig. 4  A,B). The NMA diagram was drawn in Stata 15.1 software. No link indicated the absence of direct comparative evidence. The size of treatment nodes was weighted by the number of patients, while the width of the edges, each representing a pairwise comparison, was weighted by the number of studies.

**Figure 4:**
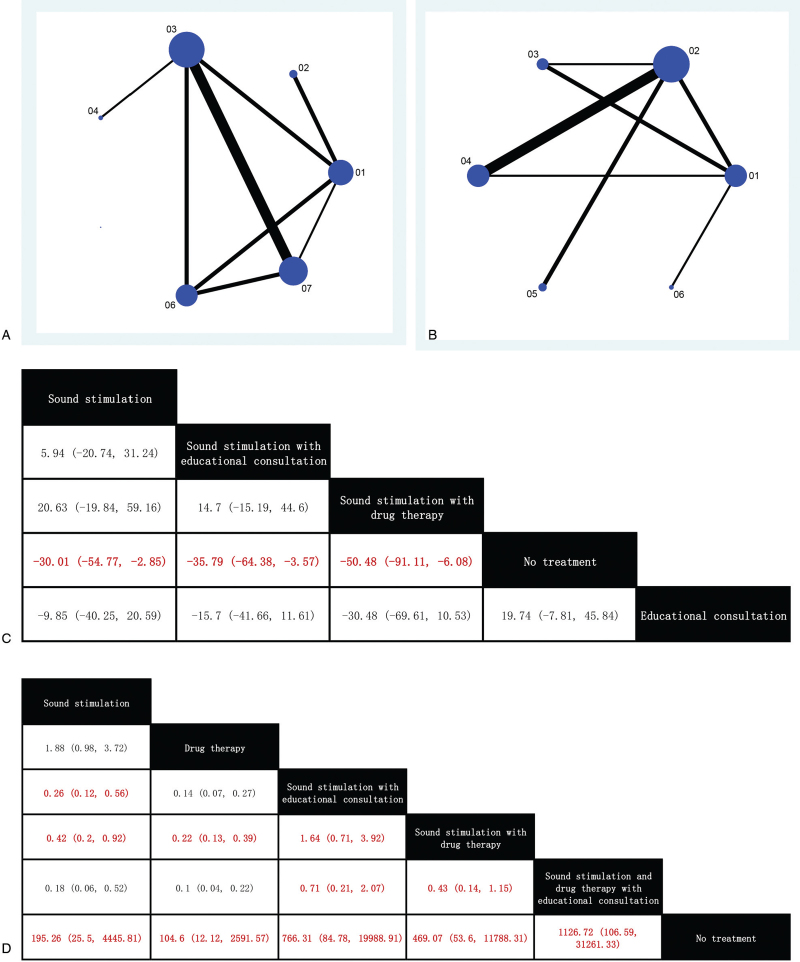
Network diagram of the change in THI score (left) and effective rate (right) (A,B); league table of the estimates of pairwise differences (C,D); intervention ranking with regard to changes in the THI scores and effect rate (E,F); Funnel plot of the studies reporting the changes in the THI score (left) and the effective rate (right) (G,H) Abbreviations: 1, Sound stimulation; 2, Drug therapy; 3, Sound stimulation with educational consultation; 4, Sound stimulation combined with drug therapy; 5, Sound stimulation combined with drug therapy and educational consultation; 6, No treatment; 7, Educational consultation. THI = Tinnitus Handicap Inventory.

**Figure 4 (Continued):**
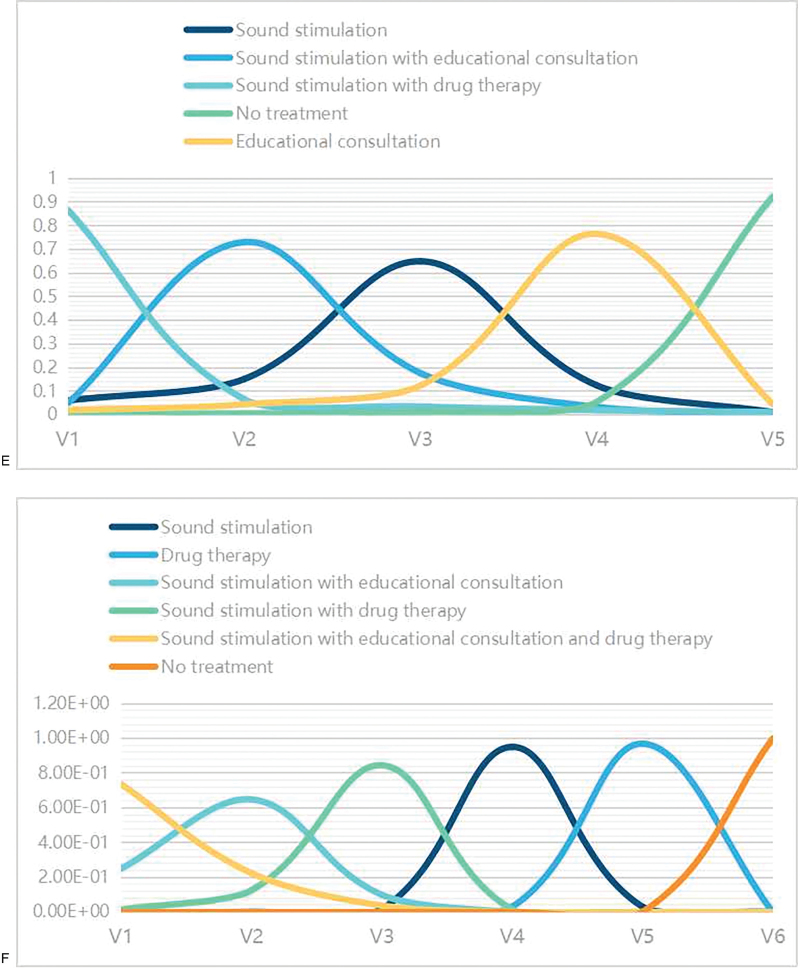
Network diagram of the change in THI score (left) and effective rate (right) (A,B); league table of the estimates of pairwise differences (C,D); intervention ranking with regard to changes in the THI scores and effect rate (E,F); Funnel plot of the studies reporting the changes in the THI score (left) and the effective rate (right) (G,H) Abbreviations: 1, Sound stimulation; 2, Drug therapy; 3, Sound stimulation with educational consultation; 4, Sound stimulation combined with drug therapy; 5, Sound stimulation combined with drug therapy and educational consultation; 6, No treatment; 7, Educational consultation. THI = Tinnitus Handicap Inventory.

**Figure 4 (Continued):**
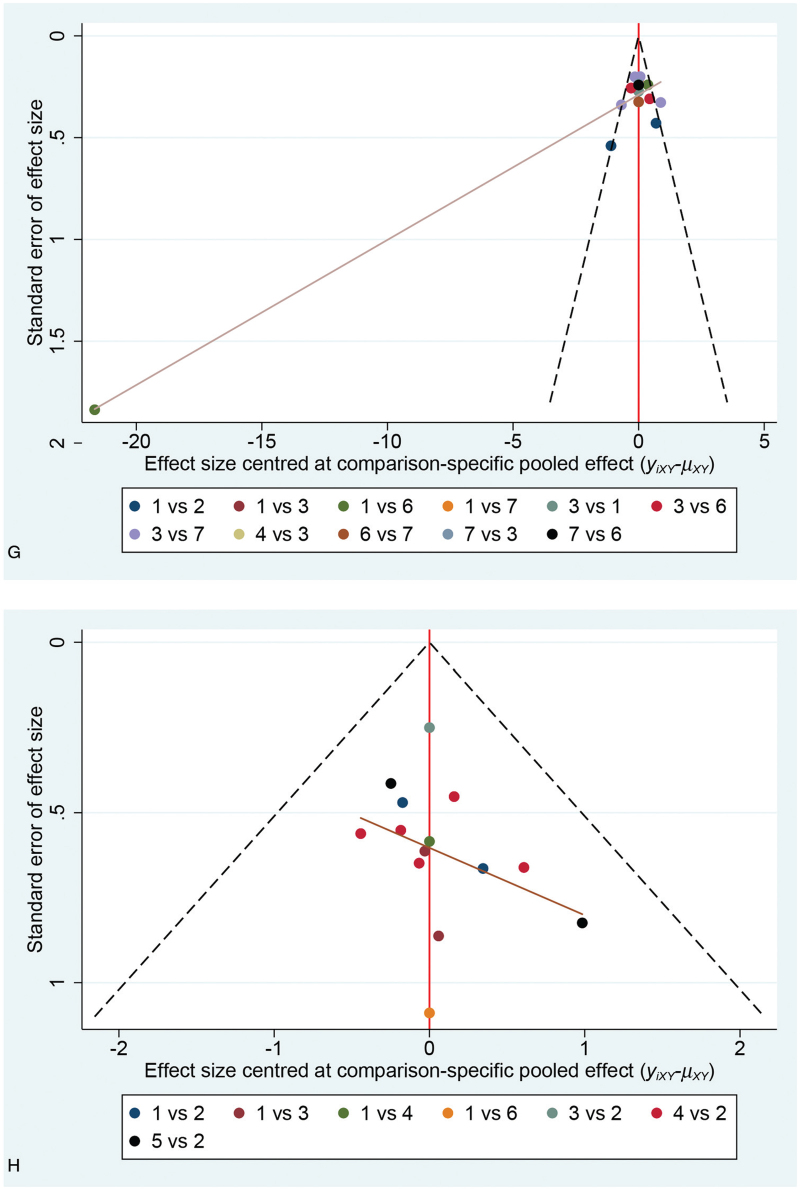
Network diagram of the change in THI score (left) and effective rate (right) (A,B); league table of the estimates of pairwise differences (C,D); intervention ranking with regard to changes in the THI scores and effect rate (E,F); Funnel plot of the studies reporting the changes in the THI score (left) and the effective rate (right) (G,H) Abbreviations: 1, Sound stimulation; 2, Drug therapy; 3, Sound stimulation with educational consultation; 4, Sound stimulation combined with drug therapy; 5, Sound stimulation combined with drug therapy and educational consultation; 6, No treatment; 7, Educational consultation. THI = Tinnitus Handicap Inventory.

#### 3.4.2 Tinnitus Handicap Inventory

Eight studies (5 arms, 668 patients) were included in the analysis of the changes in the THI score. The THI analysis included 5 sound therapy programs: sound stimulation, sound stimulation with educational consultation, sound stimulation with drug therapy, no treatment, and educational consultation.

##### 3.4.2.1 Convergence and heterogeneity evaluation

The convergence of the Bayesian model constructed in this study was evaluated in R software (version 4.0.4). The assessment showed that the fluctuation of each Markov chain was small after 20,000 preiterations, and the iteration-varying trajectory tended to become stable, suggesting that the model has converged to the stationary target distribution and has a high degree of convergence. The trajectory of the next 50,000 iterations did not obviously deflect, which indicates that the model is less likely to converge to a local solution. According to the convergence diagnostics, the change in THI scores converged rapidly to 1 after 20,000 preiterations, indicating that the results between different chains tended to be equal and suggesting that the convergence of the model is satisfactory. The satisfactory convergence means that the total number of iterations was sufficient. Therefore, the Bayesian model constructed in this study could effectively predict the posterior distribution of the parameters. Statistical heterogeneity was assessed for the NMA using the *I*^2^ statistic and the overall *I*^2^ statistic was 8%, indicating that there was no important heterogeneity.

##### 3.4.2.2 Consistency assessment

The DIC was used to compare the difference in the degree of fit between the consistency model and inconsistency model. The DIC values of the 2 models were both relatively large, with a difference of 0.424, which means that the 2 models fit each other well, indicating a relatively stable result. Therefore, the consistency model was used. Meanwhile, we used the node-splitting method to identify the local inconsistencies with *P* values >.05 in each pairwise meta-analysis. The results indicated good consistency among the studies.

##### 3.4.2.3 Network analysis

The main results of the network meta-analysis are presented in Figure 3C. The comparison between different treatments results are listed to the left of the diagonal, the effect sizes are given as the MDs with 95% CIs. Significant pairwise comparisons are in red. Only sound stimulation, TRT, and sound stimulation combined with drug therapy performed significantly better than no treatment.

We also constructed a curve of the cumulative rank probabilities of the treatments. From the rank probability plot, sound stimulation combined with drug therapy, sound stimulation combined with educational consultation, and sound stimulation alone yielded relatively higher changes in THI scores, while no treatment and educational consultation alone yielded relatively lower changes in THI scores (Fig. 4  E).

#### 3.4.3 Effective rate

##### 3.4.3.1 Convergence and heterogeneity evaluation

We also applied a burn-in phase of 50,000 iterations after 20,000 annealing algorithms to evaluate convergence. The results showed that the group had good convergence and that the model was reliable. *I*^2^ was 0%, which indicated no important heterogeneity.

##### 3.4.3.2 Consistency assessment

A difference of 0.711 points in this group suggested that there was no important difference, and the consistency model was used. We also used the node-splitting method to find local inconsistencies, and the results indicated good consistency among the reports.

##### 3.4.3.3 Network analysis

The main results of the network meta-analysis are outlined in Figure 4  D. The effect sizes are given as the ORs with 95% CIs and the significant pairwise comparisons are in red.

The cumulative rank probability indicated that the clinical treatments ranked from most to least effective were as follows: sound stimulation with educational consultation and drug therapy, sound stimulation with educational consultation, sound stimulation with drug therapy, sound stimulation alone, drug therapy alone, and no treatment (Fig. 4  F).

### 3.5 Sensitivity analysis

The sensitivity analysis showed that the removal of any individual article did not have a significant impact on the final result. Therefore, the results of the meta-analysis are reliable.

### 3.6 Publication bias

As shown in Figure 4  G, the points were concentrated at the top of the plot, and the generally observed symmetry indicated that there was no obvious publication bias or small-sample effect. However, 1 point was identified at the bottom of the funnel plot and outside the outline. In Figure 4  H, the points were distributed symmetrically in the center of the image, and 3 points were identified at the bottom of the funnel plot which indicated that there was no significant publication bias, although there may be a small-sample effect.

## 4 Discussion

Tinnitus imposes a burden worldwide and serious complications can develop from the accompanying psychological and psychosomatic symptoms.^[15]^ Sound therapy is a non-invasive treatment with broad applicability, and almost all patients qualify for this treatment. However, there is still a lack of evidence regarding the efficacy of sound therapy. The aim of this NMA was to assess the efficacy/effectiveness of sound therapy for the treatment of tinnitus. To accomplish this, 22 RCTs with 1522 participants were analyzed. This is the first NMA on this topic. NMA was used to determine the most effective treatment for tinnitus based on not only direct comparisons in RCTs but also indirect comparisons among studies.

The results of our meta-analysis not only preliminarily confirmed that sound therapy can significantly reduce the symptoms of tinnitus but also yielded a ranking of treatments in order of efficacy. In addition, combination therapies have been demonstrated to perform better than individual treatments. To evaluate the efficacy of sound therapy as comprehensively as possible, we analyzed the total effective rate and the changes in the THI and TQ.

### 4.1 Conditions of relevant existing studies

One meta-analysis^[37]^ was conducted in 2017 on the efficacy of sound therapy and conventional medical therapy for the treatment of tinnitus, and the results indicated that sound therapy led to a significantly greater overall reduction in the THI score (*X*^2^ = 2.92; df = 2; *P* = .23; *I*^2^ = 31%; *P* < .0001) and visual analog scale score (*X*^2^ = 0.25; df = 1; *P* = .62; *P* = .01) than drug therapy. The results for the total effective rate were similar (OR = 4.72; 95% CI, 3.45, 6.47; *P* < .00001). However, that meta-analysis included non-randomized controlled studies and low-quality studies, thereby decreasing the reliability of the findings.

### 4.2 Strengths and weaknesses of the study

This NMA has several strengths. First, to the best of our knowledge, this is the first NMA evaluating the evidence base for the use of different sound therapies for the treatment of tinnitus. Second, the literature search was comprehensive, and the systematic review was performed according to the PRISMA guidelines and, included all relevant studies published to date. To ensure the reliability of the conclusions, we retrieved, screened, and included previously published high-quality RCTs on the use of sound therapy for the treatment.

However, the following important limitations of this study need to be considered. First, some factors, such as different (a) criteria for the effective rate; (b) educational consultation programs; (c) frequencies and durations of treatment; and (d) types of sound stimulation, including masking therapy, music therapy, and customized sound therapy among the studies could have led to heterogeneity. Subgroups were not analyzed due to the small sample size and lack of detailed classification in the original literature. Second, there was heterogeneity in most of the outcomes among the included studies, which was attributed to the outcome measurements being based on the patients’ subjective experience and the lack of standardization of the outcome assessment. Third, indirect/mixed treatment comparisons and NMAs are still being developed and are of limited statistical value, even though they have a very promising future. Fourth, due to the number of high-quality original RCTs, the samples of the included studies were limited, and Chinese publications comprised more than half of the primary data, which may lead to some publication bias. However, we have conducted publication bias by funnel plots, and the results indicated that there was no obvious publication bias. Fifth, sleep disturbance has long been recognized as the single most important complaint among adults with tinnitus,^[38]^ however, we could not assess the treatment efficacy using the insomnia scale because of the limitation of the original studies assessing sleep condition.

### 4.3 Research needs

Based on the results of our study, we suggest that future studies focus on the following: (a) A standardized outcome measurement needs to be determined. (b) Categorizing and grouping the tinnitus population according to the baseline data, such as the causes and severity of the disease and age. (c) Different intervention protocols including educational consultation programs; frequencies and durations of treatment; and types of sound stimulation should be categorized and grouped for comparison. (d) Establishing standardized follow-up criteria in large sample studies to evaluate short- and long-term treatment impact and safety. (e) Multiple questionnaires should be used to more accurately capture the functional impact of disease and treatment, such as the Tinnitus Primary Function Questionnaire,^[39]^ which is focused on patients’ 4 primary reactions to tinnitus, emotions, hearing, sleep, and concentration, and it is considered responsive to treatment-related changes to scale the overall severity of tinnitus. (f) The results of the included studies are based on the various questionnaires which are subjective and maybe a source of heterogeneity. Nevertheless, in recent years, various studies have reported that tinnitus patients have elevated metabolic levels in some areas of the cerebral cortex compared with people without tinnitus, according to imaging examinations, such as Positron Emission Computed Tomography (PET), functional magnetic resonance imaging (fMRI) and functional near - infrared spectroscopy (fNIRS). Thus, these technologies may be a promising method to be used as objective measurements and criteria for the diagnosis of tinnitus and the determination of clinical efficacy to enhance the objectivity and accuracy of the results.

## 5 Conclusion

This is the first NMA comparing the efficacy of sound therapy with that of other treatments, and the results showed that sound therapy yielded the greatest reduction in tinnitus severity and improvement in quality of life. Furthermore, combination therapies such as TRT, sound stimulation with drug therapy, and sound stimulation with educational consultation were the most effective treatments for patients with tinnitus. However, more high-quality trials with large sample sizes and longer follow-up periods are needed.

## Acknowledgments

We acknowledge all the authors who contributed.

## Author contributions

**Conceptualization:** Hui Liu, Ting Yang.

**Data curation:** Xin Wang, Wen Zhang.

**Formal analysis:** Ting Yang, Yuan Shuang Yang.

**Investigation:** Ting Yang, Jin Zhang.

**Methodology:** Hui Liu, Ting Yang.

**Project administration:** Hui Liu, Ting Yang.

**Software:** Ting Yang, Yuan Shuang Yang.

**Supervision:** Hui Liu, Jin Zhang.

**Validation:** Hui Liu, Xin Wang, Wen Zhang.

**Writing – original draft:** Ting Yang.

**Writing – review & editing:** Hui Liu, Ting Yang, Ying Jia Li.
